# Approaches for Successful Implantation of Cardiac Implantable Devices in Patients with Persistent Left Superior Vena Cava

**DOI:** 10.19102/icrm.2023.14043

**Published:** 2023-04-15

**Authors:** Muhammad R. Afzal, Anish Nadkarni, Sapan Bhuta, Sadaf Chaugle, Essa Gul, Serene Z. Abdelbaki, Toshimasa Okabe, Mahmoud Houmsse, Ralph S. Augostini

**Affiliations:** ^1^Division of Cardiovascular Medicine, Wexner Medical Center at the Ohio State University Medical Center, Columbus, OH, USA

**Keywords:** Cardiac implantable devices, coronary sinus, persistent left superior vena cava

## Abstract

Persistent left superior vena cava (PLSVC) is the most common congenital thoracic venous anomaly, with 0.47% of patients undergoing pacemaker or cardiac implantable device placement found to have PLSVC. This review article describes challenges and interventions to successfully insert cardiac implantable electronic device leads into patients with PLSVC by providing multiple unique case examples.

## Introduction

**Figure 1:**
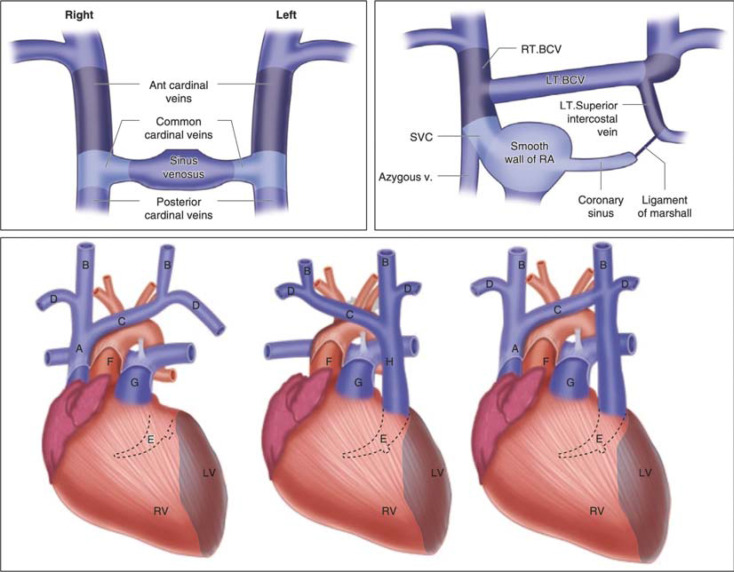
Embryological development of the superior vena cava (SVC) and variations of persistent left SVC (PLSVC). The left upper panel shows common cardinal veins into sinus venosus. The right upper panel shows that the left common cardinal vein degenerates into the ligament of Marshall. The bottom panel shows a normal development where there is the presence of a normal right-sided SVC. The middle image shows the absence of a right-sided SVC and persistence of PLSVC. The right image shows the presence of PLSVC and a normal right-sided SVC. *Abbreviations:* BCV, brachiocephalic vein; LT, left; LV, left ventricle; RT, right; RV, right ventricle; SVC, superior vena cava. Republished with permission from DeFaria Yeh D, Bhatt A. *Adult Congenital Heart Disease in Clinical Practice*. New York, NY: Springer; 2018:143–150.

Persistent left superior vena cava (PLSVC) is the most common congenital thoracic venous anomaly, with a prevalence of 0.3%–0.5% in the general population.^1^ Retrospective studies have found that 0.47% of patients undergoing pacemaker or cardiac implantable electronic device (CIED) placement have PLSVC.^2^ In the embryo, the venous system is composed of superior and inferior cardinal veins, which return blood from the cranial and caudal aspects of the embryo, respectively. These veins join to form right and left common cardinal veins before entering the embryological heart. The left common cardinal vein persists to form the coronary sinus (CS) and oblique vein of the left atrium. An anastomosis forms between the right and left superior cardinal veins during the eighth week of embryogenesis, which forms the innominate (or brachiocephalic vein [BCV]) vein. The caudal portion of the right superior vein forms the right-sided superior vena cava (SVC), and the caudal left superior cardinal vein regresses to become the ligament of Marshall. If this regression of the caudal portion of the left superior cardinal vein does not occur, it persists as a PLSVC that drains directly into the CS **(Figure 1)**. PLSVC can be categorized based on the presence of a normal right-sided SVC and bridging vein **(Figure 1)**. The most common type of PLSVC is where right and left SVCs exist. A normal innominate vein may or may not bridge. The least common subtype is the presence of PLSVC with an absent right-sided SVC.^3^ The most common drainage point of the PLSVC is the CS, which often has no hemodynamic consequences.

Most cases of PLSVC are detected as an incidental finding when a patient is seen to have a dilated CS with normal right-sided pressures on an echocardiogram. This diagnosis can be confirmed with a saline contrast echocardiogram. In patients undergoing CIED implantation, the PLSVC is often discovered incidentally during a contrast venogram performed prior to implantation of the device or when leads are inserted into the left subclavian venous system.^4^ Due to anatomical challenges, PLSVC presents unique challenges in the placement of leads for CIEDs, including cardiac resynchronization therapy (CRT) devices. Successful placement of CIEDs relies on operators utilizing a variety of techniques while adapting to the unique anatomy of the patient.

This review describes the challenges and interventions to successfully insert CIED leads into patients with PLSVC by offering multiple unique case examples.

## Case presentations

### Case 1: implantation of a dual-chamber implantable cardiac defibrillator in a patient with persistent left superior vena cava via a left pre-pectoral approach

**Figure 2:**
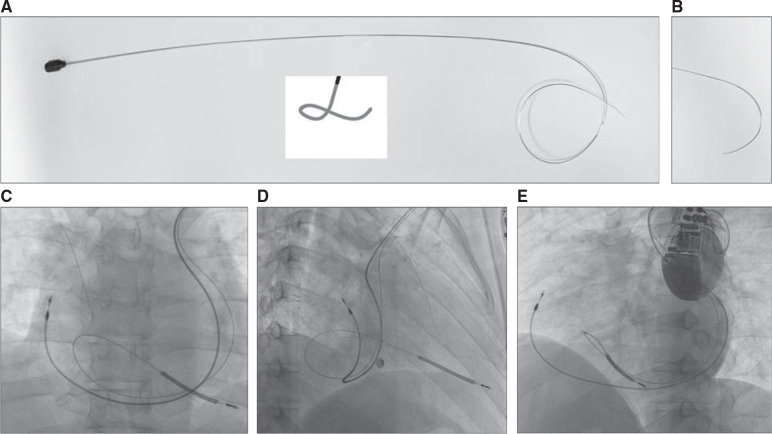
**A–E:** Implantation of a dual-chamber cardiac defibrillator in a patient with persistent left superior vena cava via a left pre-pectoral approach. **A, B:** Custom-shaped curved stylet for right ventricle (cursive L) and right atrium (broad curve) lead implantation. Final image after implantation of dual-chamber defibrillator in the anteroposterior **(B)**, right anterior oblique **(C)**, and left anterior oblique **(D)** views showing the location of the atrial lead in the right atrial appendage and the right ventricular lead in the apical septal location.

The patient is a 56-year-old man with a history of ischemic cardiomyopathy, paroxysmal atrial fibrillation, and sudden cardiac arrest who was recommended to receive a dual-chamber implantable cardiac defibrillator (ICD). A routine venogram from the left upper extremity revealed PLSVC, while a venogram from the right upper extremity revealed normal communication of the right BCV and right-sided SVC. There was no evidence of left BCV. A decision was made to proceed with the implantation of a dual-chamber ICD via the left pre-pectoral approach. A right ventricular (RV) lead was implanted first. A 65-cm single-coil ICD lead was selected. The stylet was shaped manually to assume a cursive L shape to facilitate the formation of a loop in the right atrium (RA) and entry across the tricuspid valve **(Figure 2A)**. After the lead was placed across the tricuspid valve, the stylet was fixed and the lead was advanced to reach the apical septal location. A counterclockwise movement of the stylet at the pre-pectoral pocket facilitated septal orientation of the lead. After achieving adequate pacing parameters, the stylet was retracted while slowly advancing the lead to obtain sufficient slack in the RA and RV **(Figures 2C–2E)**. For the RA lead, the stylet was custom-shaped to provide a large curve **(Figure 2B)**. A relatively longer lead (52 cm) was selected to ensure adequate slack in the RA lead. The lead was advanced through the PLSVC. The large curve of the stylet helped the lead reach the lateral part of the RA appendage, where the lead was finally implanted **(Figures 2C–2E)**. The total procedure and fluoroscopy times were 45 and 14 min, respectively. The total amount of iodinated contrast used was 10 mL for ipsilateral venography.

### Case 2: implantation of a cardiac resynchronization therapy defibrillator in a patient with persistent left superior vena cava via a left pre-pectoral approach

**Figure 3:**
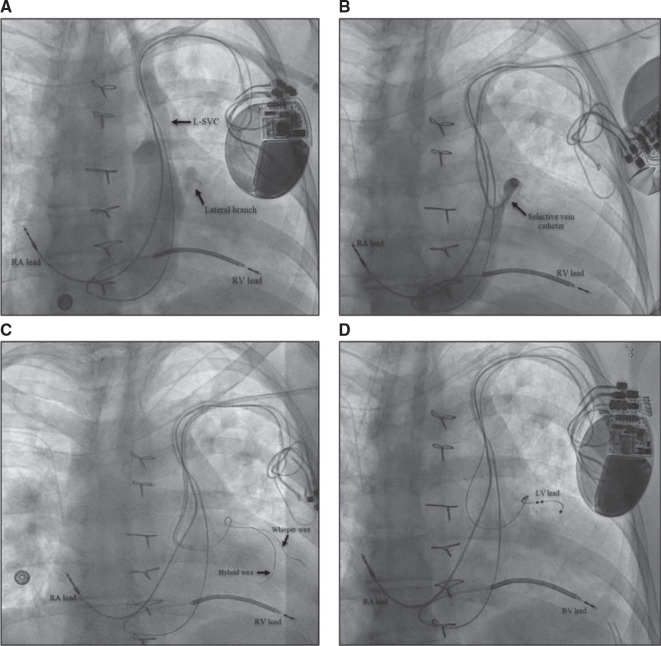
**A–D:** Implantation of a cardiac resynchronization therapy defibrillator in a patient with persistent left superior vena cava via a left pre-pectoral approach. **(A)** A venogram showing a lateral branch of the coronary sinus (CS), **(B)** use of the Worley™ vein selector to engage the CS branch, **(C)** advancing 2 wires into a CS branch in a “buddy wire” technique, and **(D)** successful advancement of CS lead into the lateral branch.

The patient was a 59-year-old woman with a history of atrial fibrillation status post–atrioventricular node ablation, tetralogy of Fallot status post-repair, pulmonary valve replacement, ventricular tachycardia with a dual-chamber ICD, and heart failure (HF) with reduced ejection fraction (EF). She was recommended to have a device upgrade to a CRT defibrillator (CRT-D) for pacing-induced cardiomyopathy with an EF of 35%–40%. The addition of a left ventricular (LV) lead via PLSVC was planned. A left arm venogram demonstrated the PLSVC with a high lateral branch of the CS **(Figure 3A)**. A selective venogram using the Worley™ lateral vein introducer (Merit Medical, South Jordan, UT, USA) demonstrated a small lateral tributary **(Figure 3B)**. A low-profile catheter capable of delivering an LV lead could not be advanced into the target branch. A “buddy wire” technique was employed using the Acuity™ Whisper wire (Boston Scientific, Marlborough, MA, USA), and an Attain™ hybrid guidewire (Medtronic, Minneapolis, MN, USA) was used in order to gain adequate wire stability into the target vein **(Figure 3C)**. A quadripolar LV lead was successfully advanced to this branch with adequate lead parameters **(Figure 3D)**. The total procedure and fluoroscopy times were 103 and 46 min, respectively. The total amount of iodinated contrast used was 45 mL for the CS venogram and intermittent injections to identify the CS branch.

### Case 3: implantation of a cardiac resynchronization therapy defibrillator in a patient with persistent left superior vena cava via the right pre-pectoral approach

**Figure 4:**
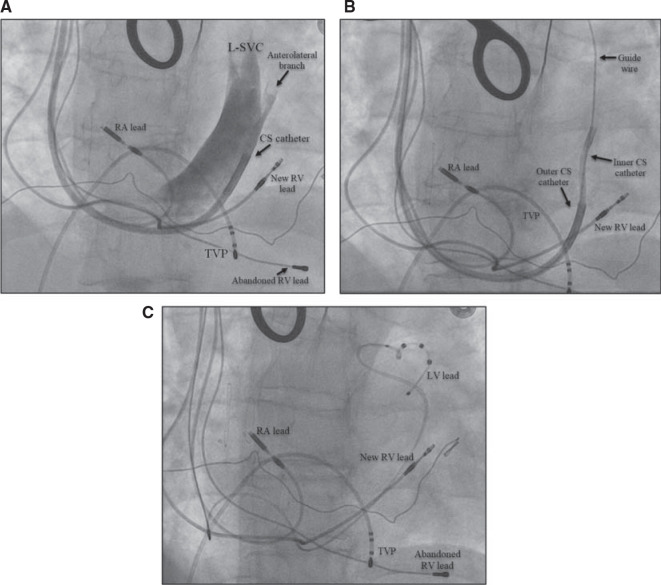
**A–C:** Implantation of a cardiac resynchronization therapy defibrillator in a patient with persistent left superior vena cava (PLSVC) via a right pre-pectoral approach. **(A)** A coronary sinus (CS) venogram showing an anterolateral branch entering into the PLSVC, **(B)** the anterolateral branch was successfully engaged with a guidewire using an inner catheter, and **(C)** successful deployment of a CS lead into a high anterolateral branch. *Abbreviations:* CS, coronary sinus; L-SVC, left superior vena cava; LV, left ventricular; PLSVC, persistent left superior vena cava; RA, right atrial; RV, right ventricular; TVP, transvenous pacemaker.

The patient was a 71-year-old woman with a history of rheumatic mitral valve disease with mitral valve repair, ventricular septal defect repair, and complete heart block implanted with a dual-chamber pacemaker. Reportedly, she had a right-sided pacemaker implanted due to “vascular issues” during a prior left-sided attempt. She developed decompensated HF, and a transthoracic echo demonstrated an LVEF of 40%–45% in the setting of chronic RV pacing. An upgrade to CRT was recommended for pacing-induced cardiomyopathy.^3^ The patient had a chronically implanted 31-year-old unipolar RV lead that was abandoned, and a new RV lead was implanted without difficulty. A CS venogram revealed a markedly enlarged PLSVC **(Figure 4A)**. The only suitable target for LV lead placement was an anterolateral branch best seen in the left anterior oblique view **(Figure 4B)**. Given the large caliber size of this side branch within a PLSVC, a Boston Scientific Acuity™ spiral quadripolar LV lead was selected as this was felt to impart the greatest probability of lead stability. Pacing from this location yielded an inferior-axis QRS complex with a negative R-wave in leads I and aVL. The final fluoroscopy revealed adequate lead positioning and pacing parameters **(Figure 4C)**. The total procedure and fluoroscopy times were 88 and 36 min, respectively. The total amount of iodinated contrast used was 60 mL for CS venography and intermittent injections to identify the CS branch.

### Case 4: implantation of a cardiac resynchronization therapy defibrillator in a patient with persistent left superior vena cava via the left pre-pectoral approach

**Figure 5:**
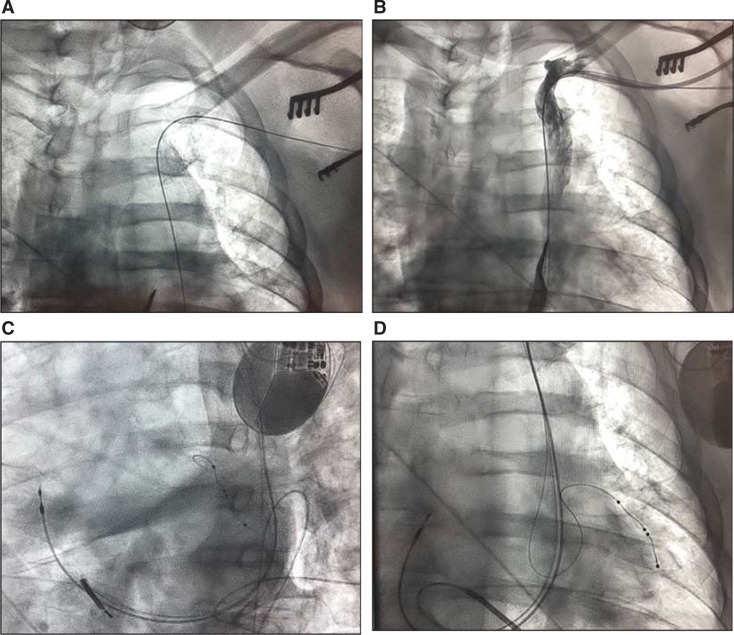
**A–D:** Implantation of a cardiac resynchronization therapy defibrillator (CRT-D) in a patient with persistent left superior vena cava (PLSVC) via a left pre-pectoral approach. **(A)** Inadvertent entry of the guidewire showing an unusual course suspicious for PLSVC, **(B)** venogram showing PLSVC, **(C)** successful implantation of the CRT-D via PLSVC in a left anterior oblique view, and **(D)** right anterior oblique view showing successful implantation of the CRT-D.

This patient was 69-year-old man with a history of non-ischemic cardiomyopathy, left bundle branch block with a QRS duration of 155 ms, and HF symptoms. The patient was recommended to receive a left-sided CRT-D. Left subclavian venography revealed PLSVC. Successful implantation of an RV ICD and an RA lead was performed as described above. A venogram of the CS revealed a large anterolateral branch. Multiple sheaths and delivery catheters with variable angulation were used to access the anterolateral branch. Ultimately, using an Attain Command™ and Attain Select™ sub-selection catheters (Medtronic), the anterolateral branch was accessed, and an Attain Ability™ (Medtronic) lead was successfully implanted **(Figures 5A–5D)**. The total procedure and fluoroscopy times were 125 and 52 min, respectively. The total amount of iodinated contrast used was 30 mL for CS venogram and intermittent injections to identify the CS branch.

## Strategies for device implantation in patients with PLSVC

PLSVC is an uncommon anomaly. Typically, this anomaly is recognized incidentally during a pre-procedural venogram or while attempting to advance device leads into cardiac chambers. Often, the left pre-pectoral pocket is already made when this anomaly is identified. Often, it is not desirable to abandon the left side at this point. The following strategies can be helpful during lead implantation in the presence of PLSVC.

### Right ventricular lead

The authors recommend using a long lead. It should be attempted to have the RV lead prolapse and form a big loop in the RA prior to the entry into the RV. As demonstrated in the first case, the RV lead has a large loop in the RA. Entry across the tricuspid valve can be facilitated by shaping the stylet into a cursive “L.”^5^ If desired, the septal implantation of the RV lead can be achieved by a slight counterclockwise rotation of the stylet or by adding a secondary curve to the cursive “L” stylet.

### Right atrial lead

An RA lead is often easy to deploy in the presence of PLSVC. After exiting the ostium of the CS, the RA lead is directed toward the lateral wall of the atrium. At this point, a stylet with a tighter curve should be used to direct the lead toward the RA appendage. Implantation of the lead into the lateral wall should be avoided to minimize the risk of lead dislodgment and cardiac perforation.

### Coronary sinus lead

Implantation of an LV lead is the most challenging part during the implantation of devices in patients with PLSVC. The cardiac venous system is not developed in the usual fashion in the presence of PLSVC. There are various approaches that have been described in the literature to overcome the difficulties encountered during the placement of CS lead in a patient with PLSVC.^6^ Some operators opt for implantation of the entire system via the right subclavian approach, while others implant only the LV lead via the right subclavian approach and then tunnel the lead to a left pre-pectoral pocket. Access to the CS from the RA may sometimes be challenging due to ostial stenosis, unroofed CS, and variable connections with the smooth part of the RA.^6^ Access to the CS in these situations may be facilitated by using an Amplatz Left 2 coronary catheter (Merit Medical) with a secondary curve to access the CS. In rare situations, tight angulation of the CS ostium can be navigated using 0.014-in guidewire via the PLSVC to the RA, where it can be snared from the femoral access for railroading the guide sheath into the CS to deliver the LV lead. After the CS has been accessed, the next challenge is finding a suitable branch. Typically, there are no cardiac veins entering into the main body of the CS. Often, lateral branches entering into the great cardiac vein are selected as a target location. Cannulation of smaller branches requires small catheters with an acute bend. After the vein is cannulated with a smaller-caliber inner, an inner sheath, or vein selectors, it is easier to advance the lead over the wire. As demonstrated in the second case, a second wire can be used as a “buddy wire” to provide stability.

## Discussion

There are multiple challenges that arise during the implantation of cardiac implantable devices in patients with PLSVC. One challenge can be attributed to patients with PLSVC having a massively dilated CS. It has been reported for the CS to be, on average, 2.55 cm in diameter.^4^ With a massively dilated CS, recording a completely occlusive venogram is often not possible. One case report specifically highlights this challenge. In this case report, a right-sided approach was initially attempted; however, the large CS precluded complete temporary venous occlusion of the CS. These operators were able to place the 3 leads using a hybrid right–left approach in which the RV and RA leads were placed using a right-sided approach and the CS lead was placed using a left-sided approach.^7^ Some operators recommend advancement of the balloon tip beyond the Vieussens valve, distal to the insertion of the PLSVC, as the vein is usually less dilated at this point and can allow for an occlusive venogram.^8^ In the rare case where a right SVC is absent, placing an LV lead through the PLSVC itself can be very challenging, as a 180° turn must be made to advance the wire past the Vieussens valve. Furthermore, the high blood flow in the area increases the risk of lead dislodgement. Case reports have shown that novel techniques such as the use of a steerable catheter-delivered lead were used to overcome an acute angle between the tricuspid valve and the CS.^9^ In this challenging scenario, a case series reported success in placing the CS lead in 2 patients with an absent right SVC using active fixation leads. Both patients had no signs of lead dislodgement and adequate function at short-term and long-term follow-up.^2,10^ Another case in this series involved this problem and was marked by multiple failed attempts to insert a defibrillator lead in the CS due to a difficult angle of access. Lead placement was successful, however, when the lead was inserted into the ostium of the middle cardiac vein. In a separate case in this case series, the difficulties of the large CS were not able to be overcome, and an epicardial LV lead was placed via a mini-thoracotomy.^11^

This case series describes the standard approaches for implantation of RA and RV leads via PLSVC. The CRT-D cases highlight the challenges associated with the implantation of an LV lead in patients with PLSVC. These cases demonstrated successful implantation from both right- and left-sided approaches. In case 1, the important factors increasing the complexity of lead placement from the left side included the tendency for the RV lead to be deflected away from the tricuspid annulus, torrential CS blood flow, acute angulation at the point where the PLSVC joins the CS, and other anomalies, making lead placement extremely difficult. In case 2, the major difficulty was maintaining stability within the CS given the vigorous blood flow from the PLSVC into the CS. In both cases, the operators were able to overcome these difficulties with equipment that is routinely used for the placement of an LV lead. The presence of PLSVC can be inferred from the presence of a dilated CS on a transthoracic echocardiogram, which is typically done prior to cardiac device implantation. If this is found and suspected, the presence of PLSVC cardiac imaging (computed tomography/magnetic resonance imaging/3-dimensional echo) can be used to understand superior venous anatomy.^12^ This may assist the operator in selecting the most appropriate approach and the necessary tools (stylets, leads, cannulation catheters, and guidewires) for device implantation, possibly increasing the chance of success.

## Conclusion

Superior venous anomalies such as PLSVC can make cardiac device implantation technically challenging. However, with increasing operator experience, cardiac imaging, and appropriate tools, successful device implantation is possible in almost all cases.
